# Supplementary material for: Are carbon emissions trading and green financial instruments synergistic? -Comprehensive quantitative research based on content analysis
Source: PLoS One. 2024 Mar 7;19(3):e0298601. doi: 10.1371/journal.pone.0298601 (PMC10919601; doi:10.1371/journal.pone.0298601)
Supplement: S1 Data — (ZIP) [file pone.0298601.s001.zip › Data/地级市Z_F工作报告.zip!/╔┬╬≈/╬╝─╧╩╨/└┤╫╘╣1⁄2╓┌║┼í╛┬φ┐╦╩2╛▌═°í┐_2018.docx]

各位代表：

　　现在，我代表市人民政府向大会报告工作，请予审议，并请市政协委员及列席人员提出意见。

　　一、2017年工作回顾

　　过去一年，是渭南加快转型、奋力赶超的一年。党的十九大胜利召开，极大地鼓舞了全市人民。在省委、省政府和市委的坚强领导下，我们深入学习贯彻习近平新时代中国特色社会主义思想和党的十九大精神，坚持稳中求进工作总基调，贯彻新发展理念，聚焦追赶超越主题，落实“五新”战略任务，加快“三地一中心”建设，较好完成了年初确定的目标任务。生产总值达到1656.62亿元，增长8.3%；全社会固定资产投资完成2731.22亿元，增长19.3%；地方财政收入实现73.58亿元，增长12%；城乡居民收入分别达到29808元和10260元，增长8.5%、9%，全市经济社会步入结构优化、提质增效的新阶段。

　　转型发展步伐加快。倾力发展新兴产业，新能源动力电池产值超过60亿元，中广核风电一期、桂花光伏并网发电。增材制造企业发展到65家。编制完成卤阳湖航空产业发展规划，国家民机试飞基地开工建设。中国酵素城入驻企业26家。规模以上工业增加值增长9.7%，非能工业产值增长31.7%。全面取缔“地条钢”，淘汰火电产能42万千瓦。着力提高农业供给质量，粮食总产211.54万吨，新增瓜果面积14.8万亩，新建扩建规模养殖场240个，我市被授予中国优质果品示范基地，白水被命名为全国绿色食品（苹果）标准化生产基地。全力创建全域旅游示范市，新增4A级景区2家，沿黄观光游成为新亮点，大荔同州湖入选国家首批体育旅游示范基地，韩城历史文化街区荣获中国最佳历史文化旅游项目。全年接待游客5300万人次，旅游综合收入426亿元，均实现30%以上增长。“陕菜之都”建设持续深化，合阳被评为中国黄河生态美食名城。“体育+”、医养结合等新业态蓬勃发展，社会消费品零售总额增长13.6%。

　　有效投资稳步扩大。始终把项目建设作为追赶超越的关键支撑，落实领导包联包抓推进机制，330个市级重点项目超额完成年度任务，中垦华山牧、龙钢百万吨带钢等98个项目建成投产，渭河综合管廊等20个重大前期项目提前开工，重点项目投资增速保持全省前列。争取中省预算内资金22.3亿元，专项债券到位41.6亿元，288个PPP项目纳入国家储备库。坚持领导干部带头招商、精准招商，举办北京经济合作活动周、上海产融结合恳谈会、杭州优势产业推介会等系列招商活动，帝亚纯电动汽车、黄土旱塬国家光伏应用领跑者基地等一批项目成功落地。全年招商引资到位资金738亿元，增长19.3%。

　　城乡面貌深刻变化。西韩城际铁路、韩（城）黄（龙）高速公路顺利开工，310国道华州段竣工通车，华山机场、西潼城际铁路前期稳步推进，大荔荣获“四好农村路”全国示范县。渭东新城加快建设，中心城市“四纵四横”道路提升改造基本完工，西海、东湖、湭河等水系建设进展顺利。渭清路综合管廊加紧建设，黄河水引入渭南北水厂。棚户区改造开工2.5万余套，货币化安置率100%，碧桂园、恒大等一批高品质住宅项目落地建设。加快补齐县城短板，华州、澄城、白水被评为国家卫生县城，潼关被评为全国首批农村幸福社区建设示范县。26个省市重点示范镇和文化旅游名镇建设完成投资48.68亿元，瓜坡镇被评为全国“一村一品”示范镇。全市城镇化率达到46.32%。全域推进美丽乡村建设，建成清洁乡村1219个、生态乡村361个，省级美丽宜居示范村15个。

　　改革创新深入推进。持续深化“放管服”改革，取消、下放、转移中省审批事项173项、市级管理权限1385项，非行政许可审批事项全部取消。推行“互联网+政务服务”，建成市级“网上办事大厅”，实施工商登记全程电子化，群众办事更加便捷。全年新登记市场主体3.9万户，增长34.5%。韩城计划单列市试点、庄里镇级市改革稳步推进，城市执法体制改革基本完成。农村“三变”改革走在全省前列，金钼独立工矿区“三供一业”分离移交试点得到国务院国资委肯定。大力推动创新创业，在西安建设“双创”孵化器，新建工程技术研究中心6家、市级众创空间7家，新增高新技术企业12家。积极推动区域协同发展，渭南高新区东区、渭南高新区蒲城园区揭牌成立，富阎一体迈出重大步伐。主动融入“一带一路”建设，设立驻外经贸代表处4个，新增友好城市2个、外贸备案企业40户，实现进出口总额11.57亿元。

　　生态建设成效明显。深入践行“绿水青山就是金山银山”理念，强力推进铁腕治霾，对所有建筑工地实行挂牌监管，先后拆改燃煤锅炉3245台，削减规上工业燃煤141万吨，淘汰黄标车829辆，大气质量持续改善。全面落实河长制、湖长制，建成生态水面1.7万亩，渭河出境断面水质稳定达标。太华湖、少华湖、蒲城洛河湿地公园纳入国家试点。深入推进“绿化秦东大地、建设美丽家园”三年行动，全年造林绿化56.6万亩。系统加强生态修复，渭北采煤沉陷区和秦岭北麓、桥山环境治理成果持续巩固。大力推进节能减排，华能秦电等10家企业实现超低排放，万元GDP能耗下降5.65%。合阳、澄城、潼关被评为省级环保模范城市，华阴被评为省级森林城市，韩城、富平被命名为省级生态园林城市（县城）。

　　民生保障力度加大。坚持把脱贫攻坚作为第一民生工程，积极推广“党支部+”产业扶贫模式，大力推进易地扶贫搬迁，1.18万户群众搬进城镇，1717户危房改造全部完工。苏陕扶贫协作持续深化，国有企业合力团对口帮扶成效初显。全年脱贫8.43万人、退出贫困村194个。全年民生支出344.1亿元，占财政支出的84.4%。新增城镇就业6.3万人，城镇登记失业率控制在3.21%。新建中小学校21所、公办幼儿园15所，临渭、华州、澄城通过国家义务教育均衡发展验收，合阳被评为国家农村职业教育和成人教育示范县。持续深化公立医院改革，全面取消药品加成，异地就医住院费用实现即时结算。大力实施文化惠民工程，成功举办全省首届全民健身运动会、国际足球赛、马拉松赛等赛事，文体活动蓬勃开展。扎实推进平安建设，全市安全生产事故同比下降52%，社会大局和谐稳定。

　　自身建设不断增强。扎实推进“两学一做”学习教育常态化制度化。严格执行中央八项规定，驰而不息纠正“四风”。加强审计监督和跟踪问效，全市“三公”经费同比下降5.61%。坚持依法行政，全面推行政务公开，政府决策更加科学，行政效能持续提升。自觉接受人大法律监督和政协民主监督，人大代表建议、政协委员提案全部按时办结。严格落实“一岗双责”，全年查处侵害群众利益不正之风和腐败问题案件1091件，处理违纪违规干部1184人。

　　国防动员、双拥工作、国家安全、人民防空、民族宗教、外事侨务等不断加强。妇女儿童、老龄、残疾人、红十字事业又有新进步，统计、气象、地震、档案、供销、地方志、新闻出版和广播电视等各项工作都取得了新成绩。

　　各位代表！过去一年，成绩来之不易。这是省委、省政府和市委正确领导的结果，是市人大、市政协大力支持的结果，是全市上下锐意进取、扎实苦干的结果。在此，我代表市人民政府，向全市广大干部群众，向各位人大代表、政协委员、离退休老同志，向各民主党派、各人民团体，向驻渭解放军指战员、武警官兵，向关心支持渭南发展的各界人士、各位朋友，表示衷心感谢，并致以崇高敬意！

　　在看到成绩的同时，我们也清醒地认识到，全市发展绝非一路坦途，依然面临着不少困难和问题。主要是：发展的质量和效益还不高，县域发展不平衡不充分的问题比较突出；传统产业转型压力较大，新的动能尚待培育，发展环境还需优化；脱贫攻坚和生态建设任务艰巨，改善民生与群众期盼还有差距；法治政府建设有待深化，“四风”顽疾仍不同程度存在，等等。对这些问题，我们一定要认真加以解决。

　　二、2018年工作基本思路和预期目标

　　2018年是贯彻党的十九大精神的开局之年，是改革开放40周年，是决胜全面建成小康社会、实施“十三五”规划承上启下的关键一年，做好今年政府工作意义重大。必须以习近平新时代中国特色社会主义思想为指导，增强“四个意识”，坚定“四个自信”；必须深刻领会省委、省政府和市委对发展形势的综合研判，提高政治站位，拓宽全局视野；必须认真审视新时代下渭南经济社会发展各项工作，明确优势潜能，抢抓赶超机遇；必须科学回答新时代对政府工作提出的“新考题”，贯彻刚性要求，增强战略定力——努力通过把握大势坚定发展信心，通过彰显优势赢得战略主动，通过激发潜能实现重大突破，砥砺奋进、担当作为，确保在跨入新时代的关键历史节点做好政府各项工作。

　　新时代的渭南，有“五大优势”更加凸显：一是政策叠加优势将进一步放大。新一轮西部大开发、“一带一路”建设有望将秦东大地发展整体推向更高能级，今年获批的关中平原城市群发展规划，与其他“国字号”政策相得益彰，更加明确了渭南在全局中的地位和作用，为全市高质量发展带来了战略机遇。二是区位交通优势将进一步聚合。陕西东大门的区位特征在全省“三个经济”的发展布局下，内涵更加丰富，围绕核心交通枢纽构建物流大通道和要素集散地的条件更加成熟，次核心地位呼之欲出，为全市高质量发展奠定了重要基础。三是产业积累优势将进一步升级。从国家三线建设时期产业初兴，到如今新型工业化加快推进，产业转型升级迈出坚实步伐，呈现蒸蒸日上、欣欣向荣大好局面，为全市高质量发展做出了物质准备。四是山水格局优势将进一步彰显。秦岭、黄河、渭水等地理标志已经成为渭南的响亮生态名片，近年来持续推进的“关中水乡”建设和大规模国土绿化，优化了生态空间，铺展了大水、大绿、大生态的美丽画卷，为全市高质量发展夯实了绿色根基。五是人心思进优势将进一步增强。处在大有可为的黄金发展期，渭南人民坐不住、等不及、只争朝夕，全市上下撸起袖子加油干，已经按下快进键、干出精气神、跑出加速度，为高质量发展提供了作风保障。

　　新时代的渭南，有“五大潜能”令人振奋：一是创新发展潜能将进一步激发。能源化工、有色冶金等传统引擎正在向高端要效益、向节能要空间，新能源汽车、增材制造、航空装备、新材料等新兴动能正在从落地到成长、从成长到聚集，双轮驱动，新旧并举，现代化经济体系加速构建，为全市高质量发展增添强劲动力。二是改革开放潜能将进一步裂变。国家机构改革逐步深入市县，“放管服”改革呈现纵深推进之势，西渭融合等协同发展机制更趋完善，优化营商环境的组合拳、连环招还要全面铺开，渭南大中小各类企业正在迎来蓬勃发展的崭新阶段，为全市高质量发展注入更多活力。三是乡村振兴潜能将进一步发掘。农业供给侧结构性改革带来结构调整、品质提升的新机遇，农村一二三产融合发展带来脱贫致富新希望，遍及市域的沃野田畴、便利交通、古朴村落更为美丽宜居乡村建设奠定基础，为全市高质量发展树立美丽样板。四是城市建设潜能将进一步转化。产城融合更加深入，城市框架不断拉大，功能配套日趋完善，承载能力明显增强，“北跨、东扩、西融、南优”的格局已经形成，必将促进城市经济发展，加速兑现城市价值，为全市高质量发展提供有力支撑。五是县域经济潜能将进一步释放。渭南各县市区县域特色鲜明，发展条件优越，各有基础，各有优势，各有招数，完全可以立足资源禀赋，推动分工协作，促进错位发展，形成一批实力强、人气旺、环境美的特色农业大县、新型工业强县、魅力旅游名县，为全市高质量发展开辟广阔空间。

　　新时代的渭南，有“五项原则”需要把握：一是坚持追赶超越。追赶超越是大局。必须在政府全部工作中提名叫响追赶超越，把追赶超越的要求明确化、具体化、常态化，横向看齐先进，纵向赶超过去，努力以闯的魄力、抢的意识、争的劲头、拼的勇气推动政府工作取得更大突破，在全省乃至更大范围的竞争中披荆斩棘、勇往直前，争先进位、勇攀高峰。二是坚持转型发展。高质量是渭南发展根本要求，现代化是城市转型根本路径。必须聚焦发展不平衡不充分问题，聚力“三地一中心”建设，高举创新旗帜，高扬改革精神，坚定不移抓重点、补短板、强弱项，倾力推进工业转型、农业提升、旅游升级、城市提质，以咬定青山不放松的韧劲，力促发展理念更新、力推发展方式转变，切实增强全市经济创新力和竞争力。三是坚持项目为王。项目工地就是阵地，项目现场就是考场，项目进度就是尺度。必须把抓项目作为经济工作最重要的方法论，着力增强项目支撑、加快项目进度，在定出节点、定准措施、定实责任上狠下功夫，满负荷运转，快节奏实施，强力度推进，努力以非凡之为、先人之举实现全市项目集群突破，支撑经济转型升级。四是坚持人民至上。人民是最大的英雄，造福人民是最大的政绩。必须把群众的利益摆在至高无上的地位，坚决贯彻以人民为中心的发展思想，强化政府是人民勤务员的意识，踏踏实实为渭南百姓谋福祉，干好事、办实事，着力补齐民生短板、促进公平正义，以人民生活的改善回馈人民给予的信任，以人民给予的信任打造让人民幸福的家园。五是坚持埋头苦干。政府的主要职责就是抓落实，抓落实就要靠苦干实干拼命干。必须增强狠抓落实本领，始终说实话、谋实事、出实招、求实效，把雷厉风行与久久为功有机结合起来，把勇于担当和善于突破有机结合起来，低调务实不张扬，以钉钉子精神做实做细做好各项工作。

　　立足新时代渭南的“五大优势”“五大潜能”和“五项原则”，按照市委五届四次全会的部署，今年政府工作的总体要求是：以习近平新时代中国特色社会主义思想为指导，全面贯彻党的十九大和十九届二中、三中全会精神，牢牢把握稳中求进工作总基调，按照新发展理念和高质量发展要求，以供给侧结构性改革为主线，全面落实“五新”战略任务，加快“三地一中心”建设，不断放大优势、激发潜能，统筹推进“八个聚力”，坚决打好防范化解重大风险、精准脱贫、污染防治三大攻坚战，一张蓝图绘到底，撸起袖子加油干，推动质量变革、效率变革、动力变革，促进全市经济社会持续健康发展。

　　2018年全市经济社会发展的主要预期目标是：生产总值增长8%以上；固定资产投资增长13%；规上工业增加值增长9%左右；地方财政收入增长9%；城乡居民收入分别增长8.1%和9%；城镇登记失业率控制在4%以内；万元GDP能耗下降3%。

　　三、2018年主要工作任务

　　今年经济社会发展任务十分繁重。必须统筹兼顾、突出重点，只争朝夕、奋勇向前，扎实做好“八个聚力”，全面推进各项工作。

　　（一）聚力重点项目建设，优化实体经济格局

　　把“项目为王”理念贯彻到发展全局、落实到具体项目，践行高质量要求，保持高强度投入，以项目服务保障建设任务，以项目引进实现发展快进，以项目带动夺取转型主动，努力为实体经济提质量、扩增量提供强有力支撑。

　　一是突出项目带动。健全重大项目推进机制，夯实领导干部包联包抓责任，抓协调、保开工、抢进度，确保350个市级重点项目完成投资980亿元。切实抓好中信重工智能机器人等170个新建项目建设，加快富平羊乳产业化改扩建等180个续建项目进度，力促金钼技改等56个项目顺利投产。切实提高项目策划包装质量和水平，抢抓国家三年滚动计划、“十三五”专项规划补充调整机遇，全年策划重大项目300个以上。

　　二是狠抓招商引资。坚持把招商引资作为“一号工程”，主动出击、以质取胜，全面推进领导干部带头招商、驻外机构定点招商、部门县市联动招商，加快构建“大招商”格局。聚焦新能源汽车整车制造和高新技术、高品质酒店、高端商业服务、优质教育医疗、精品旅游开发、农业产业化等领域，大力实施精准招商、专业招商，千方百计引进一批高精尖项目、功能提升项目。紧盯东部发达省份和行业领军企业，深化央企进渭、省企进渭战略合作，抓紧落实苏沪浙招商成果。增强“丝博会”“陕粤港澳活动周”等平台招商实效，精心筹办各类推介活动，确保全年招商引资到位资金达到780亿元。

　　三是强化服务保障。建立重点项目问题反馈和解决机制，下势破解项目建设各类难题。健全完善银企互动、企业授信机制，努力拓展项目融资渠道。降低水电气暖等要素成本，加强征地拆迁、基础设施、标准化厂房等配套保障。积极构建“亲”“清”新型政商关系，激发和保护企业家精神，严格落实产权保护政策，建立领导干部联系重点民营企业和商会制度，健全企业家参与涉企政策制定机制，千方百计帮助企业解决实际困难。我们更要坚决保护项目建设主体合法权益，对强揽工程、强买强卖、吃拿卡要等行为予以重拳整治、严厉打击，以硬茬实招让企业吃下定心丸、放手去发展。

　　（二）聚力新旧动能转换，推动工业转型升级

　　大势不可挡，狭路不可退，工业转型升级这场硬仗已经进入决战。我们要坚定不移深化供给侧结构性改革，实施创新驱动发展战略，下决心腾笼换鸟，下气力转换动能，下功夫做强支撑，决胜工业战场、跨越战略关口。

　　一是提升传统产业。实施煤炭深度转化和能源综合利用，加快推动蒲城清洁能源二期、富平燃气综合利用、陕化填平补齐等项目建设。细化“中国制造2025”渭南方案，深入推进“一企一策”，支持龙钢、青啤等企业优化资源布局、实施技术改造，扩大先进产能、推广智能制造。积极引导全市重点企业增品种、提品质、创品牌，使更多的产品迈向中高端。加大“僵尸企业”处置力度，稳妥做好企业兼并重组，盘活要素资源，腾出发展空间。

　　二是壮大新兴产业。支持沃特玛、天臣、星美等新能源电池企业加快发展，力促扩能续建项目建成投产，推动帝亚纯电动车整车下线，实现申华新能源专用车项目全面开工。大力发展光伏产业，快速推进黄土旱塬光伏应用领跑者基地建设，确保年内建成并网，带动组件制造、充电桩等关联产业协同发展。抓好中国酵素城22个项目建设，确保年内投产达效。以航空产业配套为突破，促进军民融合，稳步推进卤阳湖航空装备制造、航空小镇等项目建设，培育壮大航空产业。支持增材制造技术赶超，加大推广应用，形成产业集群。新兴产业增加值增长13%以上。

　　三是加快园区升级。充分发挥空间优势，提升产业规划和园区建设水平，积极承接东部地区和西安产业转移。支持渭南高新区加快转型升级，实现争先进位，年内启动渭河化工、金钼化学两家企业搬迁，确保2020年底前迁出主城区。加快渭南高新区东区建设，规划建设金钼工业园。推动渭南高新区与蒲城高新区协同创新，做大做强渭北煤化工业园。实施园区提档工程，支持渭南经开区、韩城经开区晋升国家级经开区。做精做专县级工业园、农业产业园，支持澄城工业园、合阳经开区、白水科技园晋升省级园区，打造县域产业园区“升级版”。

　　四是完善创新体系。设立科技创新发展基金，推动大众创业万众创新，促进创新成果转化应用。支持高等院校在渭设立双创示范基地和科技成果转化中心，建成西安创新创业孵化器。鼓励企业提升自主创新能力，年内培育高新技术企业5家、科技型中小企业20家、规模以上工业企业50家。弘扬“工匠”精神，实施专利创造质量提升工程，积极争创国家级和省级质量奖。引导金融与科技对接融合，支持优质创新型企业上市融资。加快人才公寓建设，制定更加开放便捷的人才政策，传承爱才重才的历史传统，续写代有英贤、人才济济的时代荣光。

　　（三）聚力乡村振兴战略，打造农业农村样板

　　推动农业农村现代化，渭南大有可为，理应走在前列。我们要科学制定乡村振兴战略规划，健全城乡融合发展体制机制，加快推动结构调整、体制优化、形象提升，努力打造农业强、农村美、农民富的乡村振兴渭南样板。

　　一是做强特色现代农业。抓好农田水利设施建设，加强新技术、新工艺、新装备推广应用。实施优质粮食工程，划定粮食功能区600万亩，确保粮食生产能力稳定在205万吨。实施“果畜菜”产业振兴计划，新增水果、瓜菜面积5万亩以上。抓好奶山羊全产业链培育和百万头生猪项目，新建标准化养殖场200个以上。支持苗木花卉等特色产业发展，苗木花卉经营面积达到37万亩。深化“三品一标”认证，强化农产品质量全程监管。深入实施水果公用品牌提升计划，实现精品优品直营店一线城市全覆盖。积极创建省级现代农业产业园，鼓励县市建设一批农产品深加工聚集区、田园综合体。加大休闲农业、乡村旅游、农村电商等新产业新业态培育力度，促进农村一二三产融合发展，让更多农民分享产业增值收益。

　　二是深化农村综合改革。衔接落实好第二轮土地承包到期后再延长三十年政策，探索宅基地所有权、资格权、使用权“三权分置”，推动土地入股、土地流转、服务带动等适度规模经营。稳步推进农村集体产权制度改革，全面完成清产核资任务，扩大县级农村产权流转交易市场覆盖范围。全域推进“三变”改革。引导城市资金、技术、人才、管理等要素向农村聚集，鼓励工商资本有序参与乡村振兴。实施新型农业经营主体、新型职业农民培育工程，健全农业生产全程社会化服务体系，推动小农户与现代农业发展有机衔接。

　　三是加强美丽乡村建设。分类抓好镇边村、中心村、特色村建设，逐步消除空心村。积极推进“四好”农村路建设，实施通村通组沥青（水泥）路和安全饮水巩固提升工程，改善提升65.5万农村群众饮水条件。开展农村人居环境整治三年行动计划，全域推进“厕所革命”“污水垃圾革命”，深入实施秸秆、畜禽养殖废弃物资源化利用，创建美丽宜居示范村72个。健全自治、法治、德治相结合的乡村治理体系，在持续推进移风易俗的同时，传承和保护东府乡村厚重的人文传统，守护好这片土地的根脉，滋养好渭南精神的源头。

　　（四）聚力全域旅游发展，培育全新消费热点

　　坚持以山水人文为纲，以全域推进为要，积极打造全景式覆盖、全产业发展、全季节体验、全社会参与的旅游龙头产业，带动全市服务业提质增效，促进新业态繁荣成长。

　　一是抓好全域旅游。制定实施全域旅游发展规划，抓好全域旅游示范市和高A级景区创建，支持华阴、韩城、大荔创建国家全域旅游示范区。启动华山景区提升工程，加大华山山下开发力度。推进富平爱国主义教育基地、渭华起义教育基地、沿黄公路旅游带、桃花源等特色景区提升，加快蒲城槐院里、潼关古城、合阳莘国水城、白水仓颉中华上古文化园等景区建设，建成3A级以上景区6家。完善旅游要素，大力发展户外运动、水上运动、航空运动等新业态，形成旅游新热点。支持华旅集团参与黄河金三角区域和市域内景区建设，开展联合营销、景区托管和项目开发，全力优化“两日游”“三日游”精品线路。力争全年接待游客5500万人次，旅游综合收入突破450亿元。

　　二是提高服务业层级。落实《加快发展现代服务业实施意见》。优化提升传统商贸业态，合理布局商业聚集区建设，鼓励县城建设城市综合体。持续推进“镇超”工程，建设一批农村综合超市和便利店，激发农村消费潜力。唱响“陕菜之都”品牌，完善地方特色菜系，推动餐饮服务业提档升级。加强农信社系统管理，积极引进股份制银行、股权投资、资产管理、信托、融资租赁等机构，活跃金融市场。力争新增限上服务业企业50家，全社会消费品零售总额增长11.5%以上。

　　三是培育新兴业态。支持蒲城、白水创建电子商务进农村综合示范县，实现县级电子商务服务中心全覆盖，新建200个贫困村电子商务服务点，全年电子商务网络销售额达到25亿元以上。大力发展体育产业，促进“体育+”融合发展。积极发展健康养老、教育培训等新型服务主体，鼓励社会资本投资建设一批医养中心和养老机构。设立文化产业发展专项基金，促进文化与旅游、科技、金融等融合，支持文化产业园区（基地）、文化龙头企业发展。在中高端消费、共享经济、现代供应链等领域培育新的增长点。

　　（五）聚力营商环境提升，释放改革开放红利

　　营商环境就是生产力、代表竞争力、体现创新力。我们要扎实开展“营商环境提升年”活动，全面深化改革，着力扩大开放，积极发展枢纽经济、门户经济、流动经济，加快形成活力彰显的陕西东大门。

　　一是深化“放管服”改革。进一步压时限、缩环节、减前置、优流程。加快推进“多证合一、一照一码”“证照分离”等商事制度改革。全面实施市场准入负面清单制度。持续推动市县镇村四级便民服务中心标准化建设，大力推行“互联网+政务服务”。探索推行容缺受理、上门服务等新模式，让群众办事“最多跑一次”。创新和加强事中事后监管，推行“双随机、一公开”，加快建立守信联合激励和失信联合惩戒机制，设立市级社情民意调查中心，努力做到监管更准、服务更优。

　　二是抓好重点领域改革。持续推进国资国企改革，支持市产投、城投等完善现代企业制度，组建市交投、文投等国有企业，提升主业核心竞争力。稳妥有序推进国有企业混合所有制改革。扎实推进金钼独立工矿区试点工作，全面完成省上下达的“三供一业”分离移交任务。深化庄里镇级市、农村综合试验区等重点领域改革。不断完善公共资源交易平台，加快推进盐业、供销社、财政金融、国有林场等行业领域改革。

　　三是推动“三个经济”发展。积极嫁接陕西自贸试验区政策，健全渭南口岸海关大通关联动机制，推动铁路、公路和航空等多式联运，增强面向“一带一路”的资源配置能力。建设渭南保税仓库和外贸加工园区。加快经开区渭南商贸物流园、卤阳湖通用航空和潼关物流港等项目进度，力争广汽西北商贸物流中心成功落地。推进晋陕豫黄河金三角承接产业转移示范区建设，促进企业总部、国内外客商聚集。鼓励优势企业、特色产品开拓国际市场，全年进出口总额增长10%以上。积极完善综合交通网络，加快西韩城际铁路、合铜高速、蒲白高速等项目建设，力争韦罗高速主体贯通。完成西安-潼关、渭南-蒲城-白水轨道交通和富平永济高速、秦晋黄河大桥等项目前期工作。新开工209省道渭河特大桥等7个项目，确保310国道罗敷-少华山、禹门口黄河大桥建成通车。推动华山机场全面开工，启动渭南火车站、蒲城东站提升改造，加快韩合机场和卤阳湖国家民机试飞基地建设进度。要让基础设施建设成为渭南的新亮点，着力塑造城市发展“骨骼”，打通融合发展“脉络”。

　　（六）聚力市域统筹发展，融入区域重大战略

　　全面对接国家级城市群建设布局和关中协同创新发展，坚持市域统筹、促进板块联动、加强网络化建设，同步提高城镇质量，力争全市城镇化率达到48%以上。

　　一是强化区域协同发展。全面落实关中平原城市群发展规划，围绕西安国家中心城市建设，加快西渭融合，力促富阎产业合作园高点起步、快速推进。加强与周边城市合作，加快城市间互联互通，建设关中平原城市群次核心城市。制定实施市域协同发展规划，推动中心城市、富阎、韩合、大荔、蒲白澄、大华山等六大板块内聚成核、协同共进。

　　二是提升中心城市品质。全年安排中心城市城建项目203个、投资313亿元。积极推进城市内外环线建设，完成华山大街、东风大街提升改造，打通一批“断头路”，新建一批停车场。下大气力解决主城区供水、供热、供气问题。开展海绵城市试点，通过两年努力，彻底解决城市内涝、实现雨污分流。高标准建设渭东新城、滨河新区和市民中心西片区，推动城市候机楼、五星级酒店和特色街区建设。加大棚户区、城中村和老旧小区改造力度，新建一批舒适优美、功能完善的现代社区。实施美化、亮化、净化工程，建成渭清公园、东湖、西海、南湖水系，对城市主要建筑物、所有道路实施全面亮化，持续提升保洁水平。推动城市人性化、规范化、精细化、数字化管理，拆除违法户外广告及牌匾标识，大力开展交通秩序整治活动，让城市更舒适、更宜居。

　　三是加快城镇扩容提质。积极推行“多规合一”，完成新一轮县城总体规划修编。支持韩城建设黄河沿岸区域性中心城市，推进蒲城撤县设市。实施县城提质工程，引入智慧城市、现代田园都市等发展理念，着力完善县城道路、电力、通信等基础设施，打造各具特色、环境优美的新型县城。持续抓好26个省市重点示范镇、文化旅游名镇建设，加快培育芝川、洽川2个沿黄特色小镇，充分发挥示范带动作用。

　　（七）聚力生态环境保护，推进美丽渭南建设

　　推动绿色发展是一场深刻革命。我们要坚持“绿水青山就是金山银山”的理念，把生态文明建设摆在更加突出的位置，坚决打好污染防治攻坚战，统筹推进山水林田湖草一体化治理，努力建设天蓝地绿水清的美丽渭南。

　　一是解决环境突出问题。铁腕推进“治污降霾·保卫蓝天”行动，严格实施“1+10”工作方案，强化联防联控联治，着力减煤、控车、抑尘、治源、禁燃、增绿。推进煤改气、煤改电工程，开展“电采暖”示范小区试点，扩大清洁能源覆盖面，确保空气质量持续改善。全面落实河长制、湖长制，推动管理常态化、网络化，加快园区、城镇污水处理设施建设运营，确保河流水质达到Ⅳ类标准。以更大力度推进绿色发展，坚决摒弃“黑色增长”，坚决制止和惩处破坏生态环境行为。严格项目准入，市域内不再新建火力发电厂。

　　二是推进生态保护修复。科学划定生态保护红线、永久基本农田、城镇开发边界三条控制线，编制秦岭北麓渭南段生态保护规划。深入实施生态系统保护和修复工程，加强地质灾害防治，持续抓好采煤沉陷区、秦岭北麓和桥山环境治理。加强低效用地提升改造、沟坡综合整治、工矿废弃地复垦利用，提高土地利用效率。实施最严格的水资源管理制度，加快黄河湿地省级自然保护区和国家湿地公园试点建设，促进生态系统功能提升。

　　三是狠抓柔性治水和国土绿化。加快渭南水系建设，推进卤阳湖水系、黄河西岸综合治理、城市水面等重点工程建设，新增水面8000亩，加快实现水润秦东目标。大力开展新一轮国土绿化，推进秦岭浅山区和黄渭洛河沿岸等重点区域绿化，全面实施铁路公路沿线、城市街道、村庄增绿工程，全年新增造林绿化50万亩。总之，我们要倍加珍惜渭南得天独厚的山水生态、山水格局、山水气派，实行最严格的保护措施，让大水大绿的良好生态成为百姓美好生活的支撑点、成为转型发展的生长点、成为展现渭南美丽形象的发力点。

　　（八）聚力保障改善民生，增进秦东百姓福祉

　　增进民生福祉是发展的根本目的、是不变的价值追求，必须全面贯彻以人民为中心的发展思想，深入开展脱贫攻坚，协调发展社会事业，切实促进社会和谐，倾力倾情改善群众生活。

　　一是扎实完成精准脱贫任务。积极实施“八个一批”，注重扶贫与扶志、扶智相结合，突出产业扶贫、易地扶贫搬迁、健康扶贫三项重点，全面兑现就业、教育、危改、生态扶贫和兜底保障各项政策。抓好基础设施三提升、“两房”建设和特色产业培育，大力推行“党支部+”模式，壮大农村集体经济，增加贫困群众收入。高度重视偏远落后地区和深度贫困地区群众生产生活，加大5个国定贫困县和6个深度贫困村扶贫力度，开展重大扶贫工程和驻村入户帮扶。积极对接落实苏陕扶贫协作项目，充分发挥国企扶贫合力团帮扶作用，构建各方协作抓扶贫格局。全年脱贫10.82万人、退出贫困村271个，蒲城、富平、澄城、合阳4县实现脱贫摘帽。

　　二是协调发展各项民生事业。积极拓宽就业渠道，做好大学生、进城农民工就业，全年新增就业5万人。坚持教育优先发展，加强教师队伍建设，努力解决“大班额”“择校热”、中小学课外负担重、婴幼儿照护等突出问题，年内新建改建城区中小学校13所，确保普惠性幼儿园占比达到70%。加快完善农村寄宿制学校设施，推动城乡教育均衡发展，确保大荔、富平、蒲城通过国家义务教育均衡验收。持续深化公立医院综合改革，实施基层卫生能力提升三年计划，加强中医馆和全科医生队伍建设，抓好市儿童医院规划建设。实施全民参保计划，加快医保支付方式改革，扩大异地就医即时结算和单病种结算，推进城乡居民医保一体化。为全体市民免费参投政府救助责任保险。巩固提升国家公共文化示范区创建成果，加快县级公共体育场馆提升改造，广泛开展全民健身活动，承办好国内国际精品赛事。着力稳定房地产市场，加快构建多主体供给、多渠道保障、租购并举的住房制度。

　　三是切实维护社会安定和谐。坚持问题导向、需求导向、发展导向相结合，深化社会组织管理体制改革，抓好“四社联动”，加强社区网格化管理。深化“村务公开周”活动，搞好第10次村委会换届选举。全面实施“七五”普法，加快公共法律服务平台建设。切实做好民族宗教工作。持续推进“阳光信访”“责任信访”和“法治信访”建设，坚持领导包案接访下访制度，着力化解隐患矛盾。打击非法集资、网络诈骗等违规金融活动，维护金融安全稳定。强化食品药品管理，确保老百姓“舌尖上的安全”。夯实安全生产主体责任和监管责任，全面开展大排查大整治活动，坚决杜绝重特大事故发生。严密部署“扫黑除恶”专项行动，完善社会治安防控体系，全力打造“平安渭南”，保障人民安居乐业。

　　各位代表，新时代要有新气象，新使命要有新作为。今后工作中，我们决心深入落实全面从严治党要求，不断加强自身建设，全心全意为人民服务，对人民负责，让人民满意！

　　我们将坚持依法行政。政令必行，宪禁必从。必须严格遵守宪法法律，推进法治政府示范创建，按照法定权限和程序行使权力、履行职责。坚持依法科学决策，全面落实重大行政决策程序和规定，提高决策的公众参与度。自觉接受人大依法监督、政协民主监督和社会监督，高质量办好人大代表建议和政协委员提案，推进决策科学化、民主化、法治化。

　　我们将切实改进作风。事辍者无功，耕怠者无获。必须坚持干字当头、实字为先，对中省和市委决策部署，不折不扣、坚决贯彻落实；对确定的目标任务，扭住不放、坚决推进到位。始终想群众之所想，急群众之所急，做人民的勤务员、当企业的“店小二”，认真解决各界关心的热点难点问题。全面落实“三项机制”，以更大力度激发干部干事创业活力，努力扛起时代重任、展现应有担当。我们更要时刻自警自省，“不要假装很努力，因为结果不会陪你演戏”，始终真抓实干，少说多干，让人民来检验、请历史去评判！

　　我们将加强廉政建设。清者莅职之本，俭者持身之基。必须树立廉洁奉公的事业心和勤政为民的责任心，扎实推进“两学一做”常态化制度化，认真开展“不忘初心、牢记使命”主题教育，严格遵守政治纪律和政治规矩。持之以恒落实中央八项规定和实施细则，严防“四风”问题反弹回潮。认真落实“一岗双责”，健全廉政风险防控机制，强化公共资源交易管理，加大审计监督力度，严控“三公”经费支出。坚持无禁区、全覆盖、零容忍，严惩侵害群众利益的不正之风和腐败问题，树立为民务实清廉新形象。

　　各位代表，幸福都是奋斗出来的！进入新时代，如虹征途在前、千钧重任在肩。让我们更加紧密地团结在以习近平同志为核心的党中央周围，在省委、省政府和市委的坚强领导下，不忘初心、牢记使命，攻坚克难、砥砺奋进，为谱写渭南追赶超越新篇章、创造幸福美好新生活而努力奋斗！
